# When to create embryos or organoids for research

**DOI:** 10.1136/jme-2025-110821

**Published:** 2025-07-17

**Authors:** Sebastian Porsdam Mann, Julian Savulescu, Brian D Earp

**Affiliations:** 1 Center for Advanced Studies in Bioscience Innovation Law (CeBIL), University of Copenhagen, Copenhagen, DK; 2 Centre for Biomedical Ethics, National University of Singapore Yong Loo Lin School of Medicine, Singapore; 3 Oxford Uehiro Institute, University of Oxford, Oxford, UK

**Keywords:** Embryo Research, Embryos and Fetuses, Ethics, Research, Moral Status

## Abstract

The development of brain organoids and use of human embryonic neural structures for research each raise distinct ethical considerations that require careful analysis. We propose that rather than attempting to resolve longstanding debates about embryonic moral status, a more productive approach is to examine how different positions on this fundamental question lead to distinct conclusions about appropriate research strategies. For those who ground moral status in species membership or developmental potential, even early-stage embryo research may be ethically impermissible, suggesting focus on carefully bounded organoid development. Conversely, for those who ground moral status in current capacities, embryonic neural tissue studied before the emergence of consciousness may offer significant advantages over organoids while raising fewer novel ethical concerns. Our analysis reveals inadequacies in current policies, particularly the 14-day rule, which appears difficult to justify under either ethical framework. We demonstrate how careful attention to the relationship between ethical premises and research implications can advance both scientific progress and ethical oversight, while suggesting specific policy reforms including capacity-based research guidelines and sophisticated monitoring protocols.

## Introduction

 To better understand the human brain and develop effective treatments for neurological conditions, researchers have turned to exploring various models of neural development and disease. Among these, brain organoids have emerged as a promising tool.^1^ While these three-dimensional cellular constructs derived from pluripotent stem cells offer valuable insights into neurodevelopmental processes, they face both practical limitations and ethical challenges that warrant careful examination.^2^ This paper presents an analysis of the comparative merits and ethical implications of two approaches: continued development of brain organoids versus the controlled use of human embryonic neural structures for research purposes.

We argue that one’s position on the ethical permissibility of using human embryos for research necessarily informs the evaluation of brain organoids as an alternative. If one accepts that early-stage embryo research is ethically permissible—a position that typically rests on specific assumptions about embryonic moral status and often aligns with arguments supporting early elective pregnancy termination—then embryonic neural structures may offer scientific advantages over brain organoids while raising fewer novel ethical concerns. Conversely, if one holds that embryos have significant moral status from conception, this would likely preclude both embryo research and potentially raise parallel concerns about brain organoids, particularly as they become more sophisticated.

Rather than advocating for a single position, we examine how different ethical frameworks regarding embryonic moral status lead to distinct conclusions about the permissibility and preferability of these research approaches. We proceed by first examining the practical limitations of brain organoids and the potential scientific advantages of embryonic neural structures. We then analyse how different positions on embryonic moral status inform the ethical evaluation of both approaches.

### Brain organoids and embryonic neural structures

Advancing our understanding of how the human brain functions is necessary for treating neurological and psychiatric conditions, as well as for understanding the biological foundations of cognition, emotion and consciousness. However, directly studying the living human brain presents profound ethical and practical barriers. As a result, neuroscience has historically relied heavily on animal models, particularly rodents.^3^ While these models have yielded valuable insights, the substantial differences between rodent and human brains limit their utility for understanding human-specific neural development and disease processes.^4^

Several alternatives to animal models are therefore being explored. Large-scale initiatives such as the European Union’s Human Brain Project and the United States’s Brain Research Through Advancing Innovative Neurotechnologies (BRAIN) Initiative have invested significant resources into developing computational approaches to understanding brain function. The Human Brain Project, launched in 2013, aimed to create comprehensive computer simulations of the human brain that would enable experimentation and analysis without the practical and ethical challenges of human brain research. However, despite significant recent progress in computational approaches,^5^ the extraordinary complexity of neural systems has meant that computational models remain far from capturing the full dynamics of human brain function.^6^

Another approach involves creating chimeric animals by introducing human neural cells into non-human animal brains, allowing researchers to study human neural tissue development and function in a living system (see, eg, Han *et al*).^7^ While these chimeric models have yielded valuable insights into human neural cell behaviour and integration, they face significant technical challenges in achieving sufficient human cell incorporation and raise complex ethical questions about the moral status of animals with humanised neural tissue, particularly when human cells might affect cognitive capabilities.^8 9^

Brain organoids have emerged as a third promising approach to studying human neural development and function. The promise of brain organoids lies in their capacity to model key aspects of human brain development in the laboratory, allowing researchers to observe and manipulate neural development in ways previously impossible with traditional cell culture or animal models.^10^ By generating miniature brain-like structures that mirror aspects of human neural organisation, researchers envision a future where organoids could transform our approach to studying neurodevelopmental disorders, testing new therapeutics and understanding fundamental processes of human brain formation. The ability to create patient-specific organoids from induced pluripotent stem cells also opens possibilities for personalised medicine, potentially allowing clinicians to test treatment responses before administering drugs to patients.

Brain organoids have the potential to avoid many of the practical and ethical complexities associated with chimeric models while providing a more biologically realistic system than computational approaches. However, brain organoids face their own significant limitations.^2^ They are dysmorphic, meaning that their structure fails to develop in a normal, organised way—instead of forming the precise, layered architecture seen in the human brain, they develop irregularly and chaotically. An even more fundamental problem is that they lack normal blood vessels.^1^ In a normal brain, blood vessels deliver oxygen and nutrients to cells while removing waste products. Without this vital infrastructure, organoids can only grow to be a few millimetres in size, as cells in the centre cannot receive the resources they need to survive.^11 12^ This also means organoids cannot currently develop a blood-brain barrier—the specialised boundary that controls which substances can enter the brain tissue, which is crucial for both normal brain function and for testing how drugs might work.^13^ While researchers have tried various solutions, such as growing organoids alongside blood vessel cells or using artificial fluid circulation systems, these approaches have not yet managed to replicate the complex network of blood vessels found in a natural brain.^2 13 14^

### Embryonic neural structures as superior models

Given the limitations of these three current approaches, we propose examining a fourth alternative: The use of human embryonic neural structures; that is, studying the human brain during the earliest stages of its natural development. However, rather than simply advocating for this alternative, we must first acknowledge that one’s evaluation of this proposal will necessarily depend on prior ethical commitments regarding embryo research. If one accepts that early-stage embryo research is ethically permissible—a position that typically aligns with viewing early embryos as lacking significant moral status—then embryonic neural structures may offer substantial scientific advantages while potentially raising fewer novel ethical concerns. Conversely, if one holds that embryos have significant moral status from conception, this would likely preclude such research entirely. With this caveat in mind, we examine the scientific case for embryonic neural structures as research models. We return to the ethical issues below.

Recent advances have enabled ex utero culture of early non-human primate embryos, allowing scientists to study neural tissue development outside the womb with increasing sophistication.^15^ Notably, cynomolgus monkey embryos can now be cultured from blastocyst to the neurula stage (day 25) in vitro.^15^ In such systems, the neural tube forms and neural progenitors undergo spatial regionalisation similarly to an in utero embryo, demonstrating the feasibility of studying natural embryonic brain tissue development in vitro through gastrulation and neurulation.^15^

Furthermore, research has used ex vivo human embryonic and fetal tissues obtained through other means. Fetal brain tissue from elective abortions has long been used to study human neurodevelopment.^16^ These natural tissue models offer an authentic microenvironment and the full range of embryonic cell lines, which laboratory-grown organoids strive to emulate but often lack.^16^ Organoids, for instance, can show delayed maturation or fail to reproduce the exact spatiotemporal patterning of a true embryo.^16^

Therefore, natural human embryonic neural tissue appears scientifically advantageous, particularly for studying early, organised neurodevelopment, vascularisation and processes like blood-brain barrier formation. While significant technical hurdles remain, especially for human embryo culture past 14 days,^17 18^ the scientific potential is considerable. However, it is crucial that the ethical issues associated with the use of any human embryonic tissue are addressed.

Below, we argue that if, and only if, these ethical issues can be resolved, then the use of embryonic neural structures would offer significant advantages over current alternatives, including brain organoids.

### Moral status, brain organoids and human embryos

The comparative ethical evaluation of brain organoids and embryonic neural tissue research ultimately rests on complex questions about moral status—what gives an entity moral status, to what degree and what implications this has for researchers. Following DeGrazia,^19^ Streiffer^20^ and others, we understand moral status as stemming from the possession of certain morally relevant capacities that may include, but are not limited to, consciousness, sentience, rationality, autonomy and the ability to form and pursue preferences.^9^ These capacities can exist to different extents, suggesting that moral status itself may not be binary but admit of degrees along a continuum. Entities may possess partial moral status proportionate to their possession of morally relevant capacities, rather than either full moral status or none at all.

While our analysis focuses on contrasting capacity-based views with those based on species membership or on potential development, it is important to acknowledge other approaches which we do not have space to cover. Other important frameworks feature perspectives that are likewise relevant to embryo and organoid research. Feminist ethics, for example, scrutinises implications for reproductive autonomy and the social context surrounding the sourcing of human biological materials.^21^ Communitarian perspectives^22^ may emphasise how societal values inform the perceived moral significance of potential life and the justification of research goals. Diverse religious traditions contribute specific criteria for moral status derived from concepts like ensoulment or divine creation, with different implications for research permissibility for their adherents.^23 24^ Furthermore, justice-based analyses weigh the claims of potential persons against the distributive benefits of research for existing individuals.^25^ Recognising these distinct lines of reasoning provides essential context, even as our specific contribution centres on the implications of differing foundational views of moral status.

Consciousness, for instance, is taken to be necessary on almost all accounts of moral status, except for those that base moral status on species membership alone. This understanding creates an interesting tension in evaluating the merits and ethics of brain organoids versus embryos and other research approaches. A central ethical issue in brain organoid research pertains to the possibility that these constructs could develop consciousness or sentience as they become more advanced.^26^ As organoids increase in complexity, there is a theoretical risk that they might acquire capacities warranting moral consideration akin to that afforded to sentient animals. However, this concern reveals deeper philosophical challenges about consciousness that affect both research approaches.

A fundamental challenge is the detection of consciousness from a third-person perspective.^27^ While we might identify neural correlates or behavioural indicators, determining with certainty whether an entity is conscious remains philosophically problematic—there is currently no way we can directly measure the presence or absence of consciousness.^28^ This uncertainty affects both contexts—we cannot be absolutely certain about consciousness in either organoids or early embryos. While the prevailing scientific consensus, reflected in ISSCR (International Society for Stem Cell Research) guidelines,^29^ suggests that current brain organoids do not possess consciousness, this assessment becomes increasingly uncertain as organoid sophistication advances.

In contrast, it can be argued that our understanding of consciousness development in human embryos is more definitive. Multiple lines of evidence suggest consciousness does not emerge until later in gestation, including the maturation of thalamocortical connections, the onset of coordinated neural activity and clinical observations of fetal responses.^30^ While it is difficult to make precise estimates, these factors mean that consciousness is unlikely to arise before the 24th week of gestation.^31 32^ Medical practices, such as not using fetal anaesthesia before certain gestational ages, reflect widespread confidence in the absence of early-stage pain perception.^33^ However, we must be cautious about such certainty. Historical examples—such as the medical profession’s previous belief that newborns did not feel pain—demonstrate how confident assumptions about consciousness and pain perception can be mistaken.^34^

Moreover, focusing solely on current consciousness or pain perception may miss other morally relevant considerations. Those who oppose embryo research often emphasise not just current capacities but potential—the unique trajectory of human development that, if uninterrupted, leads to a conscious person.^35^ Brain organoids, despite potentially developing some cognitive capacities, do not exhibit this developmental potential to anywhere near the same degree.

The current regulatory landscape reflects these complexities. Many jurisdictions employ the ‘14-day rule’ for embryo research,^36^ based on the primitive streak’s appearance, which is taken to signal the onset of biological individuality (ie, the first point at which it becomes clear that the embryo would, assuming the requisite conditions, develop into one, rather than two or more, individual human beings). Recent calls to extend this limit^37^ highlight ongoing debates about which developmental markers have moral significance. Meanwhile, societal practices around embryo loss—from natural miscarriage to in vitro fertilisation (IVF) procedures—suggest varying degrees of moral concern rather than absolute consensus about early embryonic moral status.^38^

However, the mere existence of such practices does not resolve the underlying ethical questions. Consider the argument that since society accepts early embryo loss in IVF and natural miscarriage, we should also accept embryo research. This logic can be reversed: if one believes embryo research is ethically problematic, this might instead suggest we should reconsider our acceptance of embryo loss in other contexts. This is what philosophers mean when they say ‘one person’s modus ponens is another’s modus tollens’—the same premises can lead to opposite conclusions depending on which beliefs we are more committed to revising.

The debate over embryo research extends beyond questions of consistency to fundamental issues about the ethical status of creating and using human life. One crucial objection centres on Kantian concerns about using human life instrumentally. Some argue that human embryos, as potential persons, should never be treated merely as means to an end but always as ends in themselves.^39^ This argument appears to preclude creating embryos specifically for research. However, this position faces several challenges. First, if we accept the use of surplus embryos from IVF for research—a practice widely permitted—we are already accepting some instrumental use of embryonic life.^40^ Such spare embryos could be used for research to replace organoid research if more useful. However, the key question then becomes whether there is a morally relevant distinction between using surplus embryos and creating embryos specifically for research. While creating embryos purely for research might seem to involve a more direct instrumental use, both practices ultimately involve using embryos as means to advance scientific knowledge and medical treatment. A more nuanced version of this objection holds that while we may use already-existing surplus embryos instrumentally, we should never create new embryos with the sole intention of using them as means.^41^ This position would still permit research using surplus IVF embryos while maintaining some restriction on instrumental creation. However, this distinction itself requires careful examination—if the instrumental use of embryos is ethically permissible in one context, the burden of proof lies with explaining why it becomes impermissible simply because that use was intended from the outset.^42^

Many people and jurisdictions hold that there is a moral distinction between intended and merely foreseen outcomes of actions, and adhere to the ‘doctrine of double effect’. On these views, it may be permissible to use spare embryos but not create embryos for research. However, this moral distinction and doctrine have been challenged, and on a consequentialist account of rightness, it will be permissible or even morally obligatory to create embryos for research and treatment.^40 42–44^

These considerations bring us back to our core thesis: rather than attempting to definitively resolve these deep ethical questions, we propose that one’s evaluation of brain organoid versus embryo research should follow from prior commitments about moral status, consciousness and potential. If one accepts that early-stage embryo research is ethically permissible, then embryonic neural structures may offer scientific advantages while raising fewer novel ethical concerns than increasingly sophisticated organoids. Conversely, if one holds that embryos have significant moral status from conception, this would likely preclude such research entirely, perhaps favouring careful development of organoid models as an alternative.

### The scientific window

The moral status of human embryos has been debated extensively, with arguments ranging far beyond the question of current capacities. Some argue that human embryos have significant moral status simply by virtue of being human (the species membership argument), while others emphasise their unique potential to develop into persons (the argument from potential).^20^ These arguments, if accepted, would likely preclude embryo research regardless of scientific benefits. Our analysis therefore applies specifically to contexts where these arguments are not considered decisive—where the moral status of embryos is judged primarily on their current capacities rather than their species membership or potential. In such contexts, we argue there exists a scientifically valuable window for embryonic neural tissue research. Within this window, we argue that embryonic neural tissue is superior to brain organoids as a model for neuroscientific and associated research.

This window would extend from the earliest stages of neural development until the emergence of morally relevant capacities. The development of these capacities appears to be gradual rather than binary. Basic neural activity begins well before any plausible emergence of consciousness or sentience, and early developmental processes—including initial cell differentiation, migration and circuit formation—occur well before consciousness could reasonably be thought to exist.^32^ This suggests that even with quite conservative estimates about the emergence of sentience, significant scientific value might be obtained from early-stage research. To clarify the boundaries of this potential research window, it is crucial to establish when developing human life attains various potentially morally relevant capacities. While this is an area of significant debate and uncertainty, currently neurobiological evidence suggests that the earliest possible potentially relevant capacity would be:

#### Rudimentary neural activity

Basic neural pathways begin functioning early, as evidenced by the development of touch sensitivity, the first sensory modality, starting around 8 weeks gestation.^32^

#### Pain perception pathway completion

Thalamocortical pathways transmitting pain signals anatomically connect to the cortex around 23–24 weeks gestation.^32 45^ However, the integrated cortical networks and processing capabilities required for conscious pain perception mature significantly later. Key indicators of this functional immaturity, such as the lack of consistent neural discrimination between painful and non-painful stimuli until the third trimester (~33 weeks), lead to the assessment that conscious pain perception is unlikely before 28 weeks gestation.^45^ Some authorities place the threshold for conscious recognition of pain later in the third trimester.^46 47^

#### Minimal sentience

Thalamocortical pathways structurally connect to the cortex around 24 weeks gestation, establishing an anatomical prerequisite for conscious sensory processing.^45 47^ However, the functional integration between thalamus and cortex, reflecting synchronised activity, strengthens significantly later, peaking between 29 and 31 weeks.^30^ Therefore, while the potential for minimal sentience following initial structural connection might be debated, robust conscious awareness is generally considered a later development (eg, from~28–32 weeks onwards), linked to increasing network maturation and wakefulness.^32 47^

This scientific window should be considered as an alternative to existing regulatory frameworks. As noted above, many jurisdictions currently employ the ‘14-day rule’ for embryo research.^36^ Recent calls to extend this limit^37^ highlight ongoing debates about which developmental markers have moral significance. However, the 14-day limit was established primarily as a practical compromise rather than based on clear ethical principles about consciousness or moral status. A capacity-based approach might suggest different boundaries—potentially allowing research by reference instead to the emergence of morally relevant capacities. One example of such a boundary might be the already-mentioned emergence of the potential for consciousness of pain at the 24th week of gestation.^31^ However, since the emergence of consciousness is likely to be a gradual rather than a stepwise process,^32^ a conservative approach would set the threshold earlier. Another potentially relevant boundary is the onset of viability, that is, the capacity to survive outside the womb. Currently, about 50% of preterm fetuses can survive without major disabilities at 22–26 weeks of development in advanced healthcare systems.^48 49^ A conservative capacity-based guideline would likely set a threshold for research well before the~28-week mark to ensure a significant buffer before the potential onset of relevant conscious experience. However, we are not attempting to argue for a precise cut-off for research—that is a subject requiring its own detailed analysis. We have merely tried to argue that up until the point at which moral status arises, embryo/fetal research is permissible on one dominant view of embryonic moral status. For the near future, the feasibility of developing embryos much past 14 days and the limits of ectogestation place practical barriers on how advanced embryonic/fetal research can be. Indeed, our arguments are premised on embryo research being of more scientific value than organoid or embryoid research. (All else being equal, if organoid research is of greater scientific utility, there is no reason to pursue embryo research.)

Crucially, many of the key considerations in this debate cannot be resolved through scientific investigation alone. While science can tell us about neural development and biological potential, it cannot determine the moral significance of species membership or the ethical weight we should give to potential future development; nor does it have much to say in relation to other proposed justifications for moral status, such as the existence of a soul. These are fundamentally normative questions, as evidenced by longstanding debates about early pregnancy termination and stem cell research. The persistence and depth of disagreement on these issues suggests that consensus may be impossible to achieve in the near term.

This is precisely why our if-then framework is valuable. Rather than attempting to resolve these deep normative disagreements, we propose examining their implications for research ethics. Those who assign significant moral weight to human potential or species membership will likely reject embryo research regardless of its scientific promise. However, for those who judge moral status primarily on current capacities, embryonic neural tissue research conducted before the emergence of such capacities may be ethically preferable to developing increasingly sophisticated organoids with uncertain capacities.

Regardless of one’s position on embryonic moral status, our framework has significant implications for the 14-day rule for embryo research. This widely adopted policy appears inadequate under either ethical framework we have examined.

The 14-day rule became influential following the Warnock Committee Report in 1984, which formed the basis for legislation in the UK. At the time, it was not possible to culture embryos longer than a few days.^50 51^ As the Chairwoman, Mary Warnock, made clear, it was a pragmatic compromise aimed at bringing together groups with polar opposite views of the embryo. It did not mark a point of moral status. ‘Instead, both in the USA and the UK, it brokered a sort of policy compromise between groups with radically different interests and views on the moral legitimacy of embryo research.’^50^

Mary Warnock stated that she chose 14 days ‘simply because everyone can count up to 14; a fortnight is a good, memorable number, and records can be kept week by week’.^51^ However, it was related to two points which were believed to have some significance.

First, the Committee was worried about the embryo experiencing pain. The neural streak, the precursor to the brain and spinal cord, appeared at day 17. It followed that the embryo before then could not experience pain. So 14 days was a safe cut-off.^50 51^ We can now be much more confident that pain perception does not begin until much later.

Second, by day 14, twinning is no longer possible. Yet the possibility of twinning has no bearing on moral status.^52^ If we acquired the power to divide, this would not affect our moral status.

There have been calls to revise the 14-day rule, despite its practical and symbolic significance.^52^ If moral status derives from species membership, developmental potential or some other non-capacities based standard, then creating embryos for research would be ethically impermissible at any stage—there is no clear ethical justification for allowing research up to 14 days while prohibiting it thereafter. Conversely, if moral status derives primarily from current capacities, then the 14-day limit appears overly conservative, potentially restricting valuable research well before the emergence of any morally relevant capacities. While the rule has served as a practical compromise, enabling some research while respecting public concerns, our analysis suggests the need for a more principled approach to research oversight or, failing that, a revision of the established compromise.

Beyond this common point, our analysis has divergent implications depending on how one grounds the moral status of human embryos. For those who view embryos as having significant moral status from conception, whether through species membership, potential or other considerations, the implications are clear: research efforts should focus on advancing organoid technology while carefully limiting their complexity to avoid the emergence of sentience or consciousness. This would require establishing clear upper bounds on organoid sophistication. Attention would need to focus on improving current organoid limitations—like vascularisation and organisation. This might also suggest greater investment in computational approaches and other alternatives that avoid both embryonic and complex organoid research.

If, however, moral status derives primarily from current capacities, then embryonic neural tissue research conducted before the emergence of such capacities offers significant advantages over current alternatives. This position would suggest extending research beyond the current 14-day limit, while establishing more sophisticated monitoring protocols and clear safety margins before any potential consciousness emergence. Such research would require robust oversight mechanisms and contingency plans should unexpected signs of advanced capacities appear. Importantly, this approach would need to maintain consistency in how uncertainty about consciousness is treated across both embryonic and organoid contexts.

## Conclusion

The comparative evaluation of brain organoid and embryonic neural tissue research reveals fundamental questions about moral status, consciousness and research ethics. Rather than attempting to resolve longstanding debates about embryonic moral status, we have shown how different positions on this issue lead to distinct but coherent approaches to advancing neuroscience research. Our analysis suggests that current policy frameworks, particularly the 14-day rule, require reconsideration to better align with either ethical position.

Looking forward, progress requires not just scientific advancement but careful attention to the interaction between empirical evidence and ethical principles. Whatever position one takes on embryonic moral status, there is a need for more sophisticated frameworks for assessing morally relevant capacities and maintaining consistent ethical standards across research contexts.

### Declaration of generative AI and AI-assisted technologies in the writing process

During the preparation of this work, the author(s) used a personalised large language model, fine-tuned on the author(s) own prior work(s), as described in,^53^ as well as Anthropic’s Claude 3.5 Sonnet, to revise a complete draft provided by JS. After using this tool/service, the author(s) reviewed and manually edited the content as needed and take(s) full responsibility for the content of the published article, in accordance with guidelines for ethical use and acknowledgement of large language models in scholarship.^54^

## Data Availability

No data are available.
